# Cell mechanical properties of human breast carcinoma cells depend on temperature

**DOI:** 10.1038/s41598-021-90173-y

**Published:** 2021-05-24

**Authors:** Christian Aermes, Alexander Hayn, Tony Fischer, Claudia Tanja Mierke

**Affiliations:** Biological Physics Division, Faculty of Physics and Earth Science, Peter Debye Institute of Soft Matter Physics, University of Leipzig, Linnéstr. 5, 04103 Leipzig, Germany

**Keywords:** Nanoscale biophysics, Biological physics

## Abstract

The knowledge of cell mechanics is required to understand cellular processes and functions, such as the movement of cells, and the development of tissue engineering in cancer therapy. Cell mechanical properties depend on a variety of factors, such as cellular environments, and may also rely on external factors, such as the ambient temperature. The impact of temperature on cell mechanics is not clearly understood. To explore the effect of temperature on cell mechanics, we employed magnetic tweezers to apply a force of 1 nN to 4.5 µm superparamagnetic beads. The beads were coated with fibronectin and coupled to human epithelial breast cancer cells, in particular MCF-7 and MDA-MB-231 cells. Cells were measured in a temperature range between 25 and 45 °C. The creep response of both cell types followed a weak power law. At all temperatures, the MDA-MB-231 cells were pronouncedly softer compared to the MCF-7 cells, whereas their fluidity was increased. However, with increasing temperature, the cells became significantly softer and more fluid. Since mechanical properties are manifested in the cell’s cytoskeletal structure and the paramagnetic beads are coupled through cell surface receptors linked to cytoskeletal structures, such as actin and myosin filaments as well as microtubules, the cells were probed with pharmacological drugs impacting the actin filament polymerization, such as Latrunculin A, the myosin filaments, such as Blebbistatin, and the microtubules, such as Demecolcine, during the magnetic tweezer measurements in the specific temperature range. Irrespective of pharmacological interventions, the creep response of cells followed a weak power law at all temperatures. Inhibition of the actin polymerization resulted in increased softness in both cell types and decreased fluidity exclusively in MDA-MB-231 cells. Blebbistatin had an effect on the compliance of MDA-MB-231 cells at lower temperatures, which was minor on the compliance MCF-7 cells. Microtubule inhibition affected the fluidity of MCF-7 cells but did not have a significant effect on the compliance of MCF-7 and MDA-MB-231 cells. In summary, with increasing temperature, the cells became significant softer with specific differences between the investigated drugs and cell lines.

## Introduction

Temperature is a key parameter in many physical, biological and biochemical processes. In the body, temperature may be increased due to diseases, such as cancer, fever, or physical activity. Elevated temperatures may influence cell morphology, motility, biochemical activity and thus cell functionality^1–3^. For example, the metabolism of cells is largely temperature dependent^4,5^. Metabolic rates increase as temperature increases, until a peak for the metabolic rate is reached. Beyond that, a further increase in temperature decreases the metabolic rate^6,7^. This may be especially important in cancer cells, that generally display an increased metabolic activity compared to healthy cells^8,9^. Hence, the elevated temperature is often observed in the malignant progression of cancer cells^10,11^ and may play a role in the mechanical characterization of cancer cells.

Even though temperature plays a crucial role in many cellular processes, the impact of temperature changes on cell mechanics is not understood in great detail. There exist studies that report a cell stiffening with increasing temperatures^12–14^, whereas others report a temperature induced softening of the cell^1,13,15–22^.

In addition, the physical heating affects cancer cells and healthy cells differently^1^. Thermoprotective mechanisms in cancer cells may be deregulated, leading to a higher rate of cell death after heat treatment compared to healthy cells in vitro^23,24^. The different response of cancerous and healthy cells to changes in temperature has inspired the development of hyperthermia, i.e. the increase of body temperature to about 43 °C, as a treatment of various cancer types in combination with conventional chemo and/or irradiation therapy. This heat treatment of cancer cells makes them more susceptible to damages from the radiation and additionally increases the cell's intake of drugs. Moreover, the damage to normal cells of the surrounding healthy tissue due to the increased temperature is minimal^25–27^. Hyperthermia has been tested successfully, for example, in the treatment of breast cancer^26,28,29^. However, the mechanism is not well understood and may be based on cell mechanical alterations.

Studies on the mechanical properties of cells have established a linkage between the specific physical properties of cancerous and healthy cells and differences in their respective structures^30,31^. These distinctive biophysical properties of cancer cells may therefore also be related to the different response to temperature changes^13^. For simplicity, most studies of cell mechanics are performed either at physiological temperatures (37 °C) or at room temperature (around 25 °C)^32,33^. Due to the effect of temperature on cells, it is essential to employ precisely controlled temperature measurements during the assessment of the cell mechanical properties of living cells^18,22^.

A systematic investigation of the effect of temperature on cellular mechanics, such as compliance and fluidity, is likely to reveal new insights into the underlying structures and cellular processes that drive cellular response to external stress and strain. Thereby, the external stress and strain is exerted on paramagnetic beads coated with the extracellular matrix protein fibronectin, which binds to cell matrix adhesion receptors, such as the α5β1 integrin. Due to the binding of the fibronectin-coated bead to the receptors, they get engaged with the cytoskeleton of the cell, such as actin filaments, myosin filaments and microtubules. In summary, the goal of our study was to analyze the effect of temperature on two distinct human epithelial breast cancer cells of different migratory capacity in 3D environments, such as MCF-7 and MDA-MB-231 cells at a temperature range covering at its higher end also the temperature employed during hyperthermia treatments. MCF-7 and MDA-MB-231 cells are commonly used model systems for breast carcinoma that show most of the abnormalities observed in breast cancer in vivo^34,35^. MCF-7 cells have been frequently demonstrated to be less invasive than MDA-MB-231 cells^31,36^, which renders them good model systems for non-aggressive and aggressive cancer, respectively. At the same time, their distinct invasive behavior has been linked to differences in their mechanical properties^30,37,38^. Both cancer cell lines differ in their cytoskeletal structure, which is manifested in their distinct mechanical properties and invasive behaviors^30,31,39^. Therefore, we aim to link specific cytoskeletal components, such as the actin and myosin filaments as well as microtubules to the observed temperature-dependent (or induced) effects on these cells by comparing their overall respective thermorheological response.

## Material and methods

### Cells and cell culture

Cells were cultured in high glucose (4.5 g/l) Dulbecco’s modified Eagle’s medium (DMEM, Biochrom, Berlin, Germany) supplemented with 10% fetal bovine serum (FBS) (Biochrom, Berlin, Germany) and 1% penicillin/streptomycin (P/S, Biochrom, Berlin, Germany). Cells were passaged at a confluence of about 80%.

### Magnetic tweezer measurements

For magnetic tweezer measurements, 4.5 µm superparamagnetic beads (Dynabeads M450, Sigma Aldrich) were coated with human fibronectin (50 µg/ml, Sigma Aldrich) following the description by the manufacturer. For this, beads were first washed with PBS. Afterwards, fibronectin was added. The beads were then centrifuged for 24 h at 37 °C and at a centrifugal force of 8×*g*. Thereafter, the beads were washed twice with PBS and stored at 8 °C. Prior to measurements, beads were rigorously agitated by a vortex mixer to break up any clusters that may have formed during storage.

For measurements, cells were prepared as described previously^40,41^. Briefly, about 24 h prior to the measurement, cells were seeded onto 35 mm culture dishes (about 10^5^ cells per dish). Cells were then incubated at 37 °C and 5% CO_2_. On the day of the measurement, about 5 ×  10^4^ fibronectin coated beads were added to the cells. The cells were then incubated at 37 °C and 5% CO_2_ for 20 min. In detail, the cells were measured no longer than 40 min. After that time, beads were usually internalized by the cells. About 30 cells could be measured during the 40-min interval^41,42^. Cells were measured at a 40-fold magnification. At this magnification, the field of view is about 0.1 mm^2^ in size. In this field of view, about 15–20 individual cells were visible.

Only cells with a single bounded bead were investigated. The bead-needle distance was chosen in such a way that the maximal possible force was greater than the desired force. When placing the needle, exceptional care was taken to avoid poking the cell with the needle tip.

For pharmacological interventions, cells were treated with different inhibitors targeting specific components of their cytoskeleton. Actin polymerization was inhibited by treatment with 0.2 µM Latrunculin A^31^. Myosin activity in the cells was inhibited by treating the cells with 25 µM Blebbistatin^31^. Microtubules were depolymerized by treating the cells with 0.08 µM Demecolcine^43^. For all treatments, cells were incubated for about one hour with the respective drugs before they were probed with the magnetic tweezer.

### Magnetic tweezer technique setup

The custom-built magnetic tweezer setup used in this study has been described previously^41^. Briefly, the setup consists of a coil with a superparamagnetic core that extends beyond the coil and has a sharp conical tip. The magnetic tweezer is connected to a 3D-micromanipulator and mounted on top of an inverted microscope (DMI8, Leica). The microscope is equipped with a CMOS camera (Orca Flash 4.0 V3, Hamamatsu). The entire setup is enclosed by an environmentally controlled chamber that allows precise control over the ambient temperature in a range between 20 and about 47 °C. Additionally, the chamber is equipped with a CO_2_ supply and a sensor to measure the concentration of CO_2_ inside the chamber. The CO_2_ flows through a water reservoir inside the chamber. The reservoir pre-saturates the gas with water and heats the gas to the ambient temperature. From this water reservoir, the CO_2_ flows through a tube and is directed at the sample. The flow of CO_2_ is automatically regulated to ensure a stable concentration of 5% CO_2_ in the chamber. The setup is controlled by a custom-written LabVIEW program for tracking of beads and automatic adaption of the coil current to ensure the application of a constant force as the bead moves closer to the tip of the magnetic tweezer.

**Figure 1:**
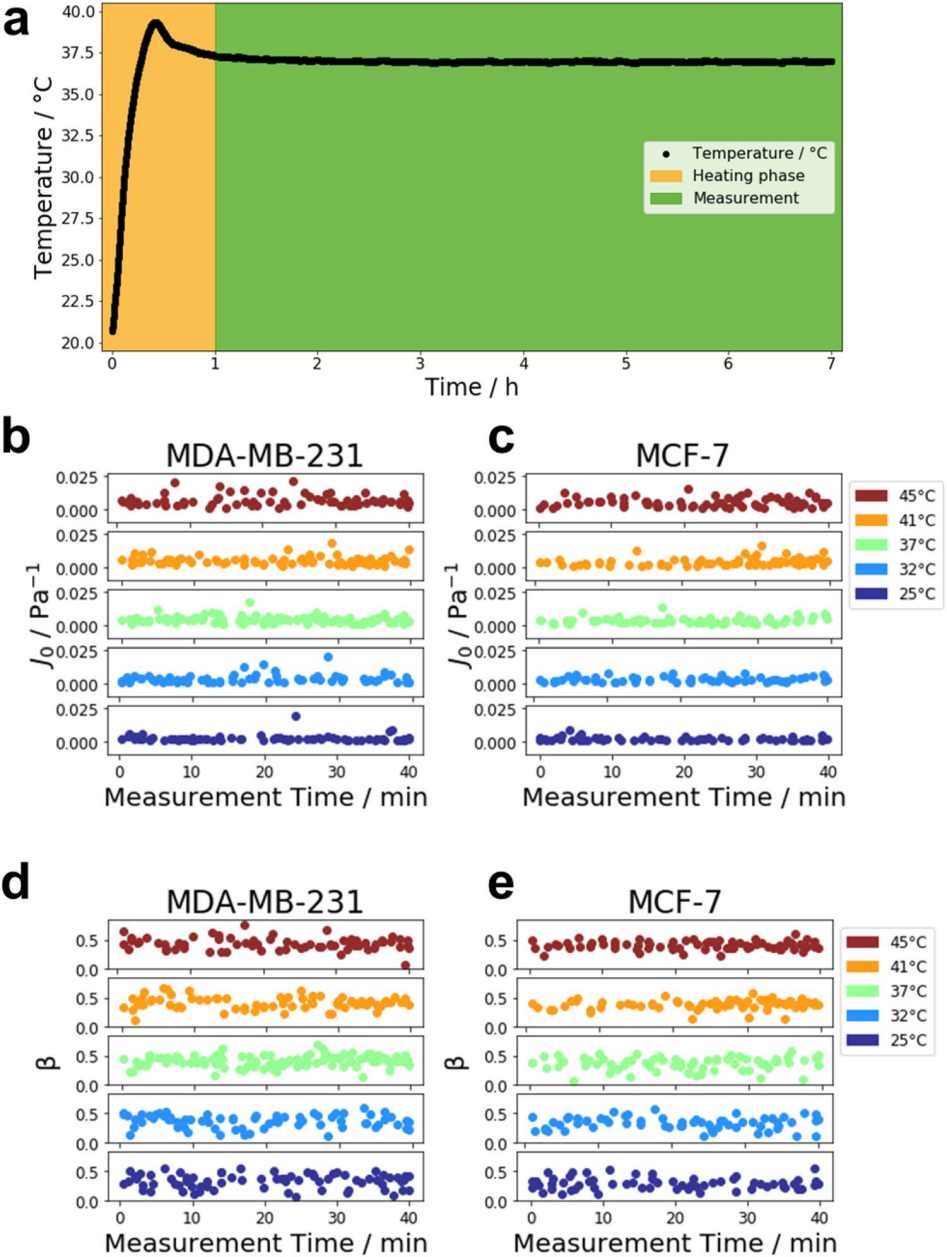
Heat stability of the magnetic tweezer system. (a) Heating curve measured inside the incubation chamber with a target temperature of 37 °C. Temperature was recorded over the duration of a typical measurement. The recording starts at the time the heating unit is switched on. The heating phase is displayed in orange and lasts for about 1 h. During the heating, the temperature inside the chamber rises drastically to a temperature slightly above the target temperature. After that, the temperature drops down to the target temperature and remains stable during the time of measurement (displayed in green). During the magnetic tweezer measurement the J_0_ value of MDA-MB-231 (b) and MCF-7 (c) breast cancer cells was not significantly altered over time and also the β value of MDA-MB-231 (d) and MCF-7 (e) cells was not significantly changed over time. This has been shown for all chosen temperatures, such as 25 °C, 32 °C, 37 °C, 41 °C and 45 ° C.

The heating unit and the CO_2_ supply were turned on about one hour before the measurement to ensure stable environmental conditions during the experiments. An exemplary heating curve for a measurement at 37 °C is presented in Fig. 1a. During the one-hour heating period, the temperature inside the chamber rises quickly and then stabilizes. During the measurement, the temperature remains stable at the target temperature of 37 °C. Cells were measured at specific temperatures, such as 25 °C, 32 °C, 37 °C, 41 °C and 45 °C. Before cells were measured, they were kept undisturbed inside of the incubation chamber for about five minutes to let the temperature of the culture medium adjust to the outside temperature. This was usually enough time to establish a thermal equilibrium due to the small volume of culture medium in the petri dish of 2 ml. Variations in the mechanical properties of cells even within the same cell line and under the same conditions may arise from the dynamic structure of the cells and differences in their morphology^44^. Additionally, the differences in the cellular response may also partially attributed to cells being in different states of the cell cycle^15^.

Cells were measured by employing a single rectangular force pulse with a magnitude of one nanonewton for two seconds. After the creep response was measured, the force was removed, and the subsequent relaxation of the cells was recorded for another 2 s. In order to retrieve cell mechanical properties from the recorded displacement curves, we calculated the compliance J(t). The creep compliance is defined as the ratio of strain ε over stress σ and is a commonly used property to describe the mechanical properties of viscoelastic materials^33,45,46^. The sheer stress experienced by the cell is equal to the ratio of the applied force ΔF and the contact area A between cell and bead. Since the images taken are two dimensional projections, the exact contact area could not be determined. However, from previous studies it is known that beads are internalized after about 40 min^41,42^. Therefore, the beads adhered only to the surface of the cell during the measurement. Hence, the contact area was approximated as A = πr^2^. This area corresponds to a bead indenting the cell with a depth of half its radius. This estimation has been used in earlier studies and leads to an expression of σ = ΔF/(πr^2^) for the applied stress. In order to estimate the strain, we divided the displacement of the bead by the bead radius, which is a characteristic length scale of the experiment. Therefore, the displacement of the beads was converted to a creep compliance by scaling the displacement curves with the applied force πr/ΔF, such that J(t) = d(t) × πr/ΔF^33,41^.

In order to retrieve the mechanical properties of the cells, the creep phase (i.e. the first two seconds) of the resulting curves were fitted with a weak power law, which has been shown to accurately characterize both localized and global rheological properties of cells over several orders of magnitude^47,48^

1 $$J\left( t \right) = J_{0} \left( {{\raise0.7ex\hbox{$t$} \!\mathord{\left/ {\vphantom {t {t_{0} }}}\right.\kern-\nulldelimiterspace} \!\lower0.7ex\hbox{${t_{0} }$}}} \right)^{\beta }.$$

Here t_0_ is a reference time which was set to t_0_ = 1 s as a typical length scale of the experiment. The parameter t_0_ is used to normalize time in order to get a dimensionless exponent. The choice of t_0_ is arbitrary and merely affects the definition of J_0_ = J(t = t_0_). The prefactor J_0_ is the creep compliance of the cell and is a measure for its stiffness. That is, J_0_ is proportional to the inverse of the stiffness^15,33,49^. The dimensionless parameter β characterizes the viscoelastic state of the cell. At β = 0 the cell behaves as an ideal elastic solid following Hooke’s law, whereas at β = 1 it behaves as a Newtonian liquid. Therefore β is a measure for the fluidity of the cells^15,38,49^.

Only the creep phase was used for the analysis. Exemplary fit curves are given in Supplementary Fig. S1. The weak power law generally fits the experimental data well. The relaxation part of the recorded curves was not fitted. However, the relaxation phase was used to filter out cells from the analysis. The expected behavior of cells after the removal of force is a slow and continuous relaxation^50^. Cells that did not display such a recovery but instead displayed a continued creep were discarded from the analysis. The continued creep was usually attributed to active processes or (partial) detachment from the substrate.

For every condition, about 60 cells were measured and analyzed. The measurements were taken on three independently prepared samples, i.e. about 20 cells per sample were measured. Average quantities were then derived as the population average from all cells measured across these multiple samples. For J_0_, the average was calculated as the geometric average, while for β the arithmetic average was used^40,41^.

### Statistical assessment

For significance tests, a Welch’s t-test was performed. Additionally, a Bonferroni correction was carried out to compensate for possible errors introduced due to multiple tests. Due to the log-normal distribution of the compliance values, the significance test was carried out on the log-transformed values. Results were considered significant for p < 0.05 and are marked by asterisks in the respective figures (*p < 0.05, **p < 0.01, ***p < 0.001).

## Results

### Thermorheological measurements of human breast cancer cells

To investigate the effect of temperature on cell mechanics of breast cancer cells, we analyzed for each temperature (25 °C to 45 °C), the mechanical properties of well-known human breast carcinoma cells MDA-MB-231 and MCF-7 cells. In specific detail, the two cell types were measured by applying a constant force of one nanonewton over two seconds. For this, the tip of the magnetic tweezer was placed at a distance between 20 and 50 µm away from a bead bound to a cell. All measurements were carried out in an environmental chamber supplied with 5% CO_2_.

The displacement of the bead was tracked over time and fitted with a weak power law.

In order to check for cell viability, the measured properties J_0_ (compliance) and β (fluidity) were plotted over the course of the measurement for both cell lines. The compliance of MDA-MB-231 and MCF-7 cells did not vary significantly over the 40 min of the measurement (see Fig. 1b,c, respectively). Similarly, cell fluidity for MDA-MB-231 and MCF-7 cells also remained stable and did not show a significant increase or decrease during measurements (see Fig. 1d,e, respectively). Cells show slight variations even within the same cell line and under the same condition. These variations may be attributed to different morphologies of individual cells and possible different states in the cell cycle^15,44^. Additionally, cell viability was judged based on the videos taken during the measurements. MDA-MB-231 and MCF-7 cells measured at the beginning of an experiment in a 25 °C environment (Supplementary Videos 1, 2, respectively) did not differ significantly from those measured at the end of the experiment (Supplementary Videos 3, 4, respectively). Similarly, at an ambient temperature of 45 °C, MDA-MB-231 and MCF-7 cells measured at the beginning of an experiment (Supplementary Videos 5, 6, respectively) did not show a changed morphology compared to those observed at the end of a measurement (Supplementary Videos 7, 8, respectively). Therefore, cells were viable over at least the 40 min it took to measure the cells both at the highest and the lowest temperature investigated.

**Figure 2:**
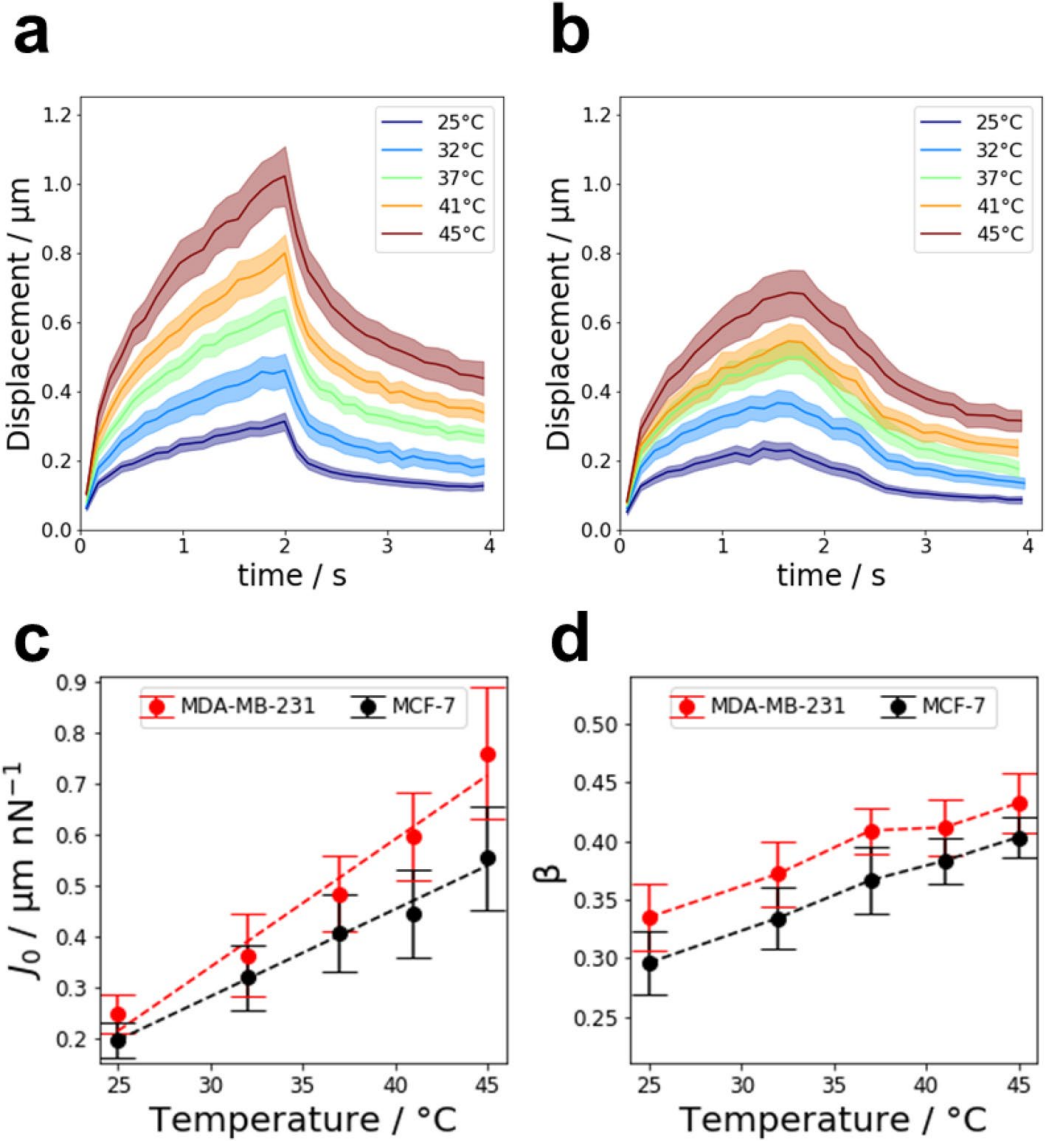
Response of MDA-MB-231 and MCF-7 cells to an increase in temperature from 25 to 45 °C. (**a**) Average displacement curves for 4.5 µm superparamagnetic beads coupled to MDA-MB-231 cells in response to a force of 1 nN. (**b**) Averaged creep curves for MCF-7 cells. The creep curves of both MCF-7 and MDA-MB-231 cells closely follow a power law. (**c**) Average creep compliance of MDA-MB-231 and MCF-7 cells as a function of temperature. The average creep compliance was derived from the pre-factor of a power law fit to the respective creep curves. For both cell lines, the creep compliance increases linearly with temperature. (**d**) Average cell fluidity of MDA-MB-231 and MCF-7 cells. The cell fluidity β is the exponent of the power law fit to the creep curves. Both cell lines become more fluid-like as the temperature increases.

The averaged displacement curves of MDA-MB-231 and MCF-7 cells are presented in Fig. 2a,b, respectively. From the fit, the average cell creep compliance and fluidity were derived. The average creep compliance of MDA-MB-231 and MCF-7 cells is presented in Fig. 2c as a function of temperature. For all temperatures investigated, MDA-MB-231 cells were significantly softer than MCF-7 cells. For both cell lines, the average creep compliance increased linearly with increasing temperature. For MCF-7 cells, the creep compliance increased by a factor of 2.8 ± 0.7 from 25 to 45 °C. MDA-MB-231 cells experienced a stronger relative softening as their creep compliance increased by a factor of 3.1 ± 0.7 as the temperature increased from 25 to 45 °C. Similarly, cell fluidity β also increased for both cell lines with temperature, i.e. cells became more viscous, as presented in Fig. 2d. Here, MDA-MB-231 showed a higher cell fluidity β than MCF-7 cells for all temperatures. The relative increase in cell fluidity from 25 to 45 °C was similar for MCF-7 and MDA-MB-231 cells, with relative increases of 1.4 ± 0.1 and 1.2 ± 0.1, respectively.

### Pharmacological intervention

In order to investigate whether a specific cytoskeletal component, such as actin filaments, myosin filaments or microtubules, is responsible for the observed temperature dependent cell compliance and fluidity, different components of the cytoskeleton were inhibited by pharmacological intervention. Since the superparamagnetic fibronectin-coated beads are connected to the cytoskeleton via cell–matrix adhesion receptors, it seems to be reasonable to reveal which types of cytoskeletal components are involved. Cells were treated separately with three different drugs targeting specific cytoskeletal components. Latrunculin A was used to inhibit the polymerization of actin. Myosin-II was inhibited by Blebbistatin, which interferes in a blocking manner with the actin-myosin head interaction. Besides the actomyosin cytoskeleton, microtubules can also provide mechanical stability for the cell^51,52^. Hence, cells were also treated with Demecolcine to depolymerize the microtubules of the cell. Control measurements were carried out by supplementing the culture medium with the same volume of dimethyl sulfoxide (DMSO) as in the measurements with the respective inhibitors.

**Figure 3:**
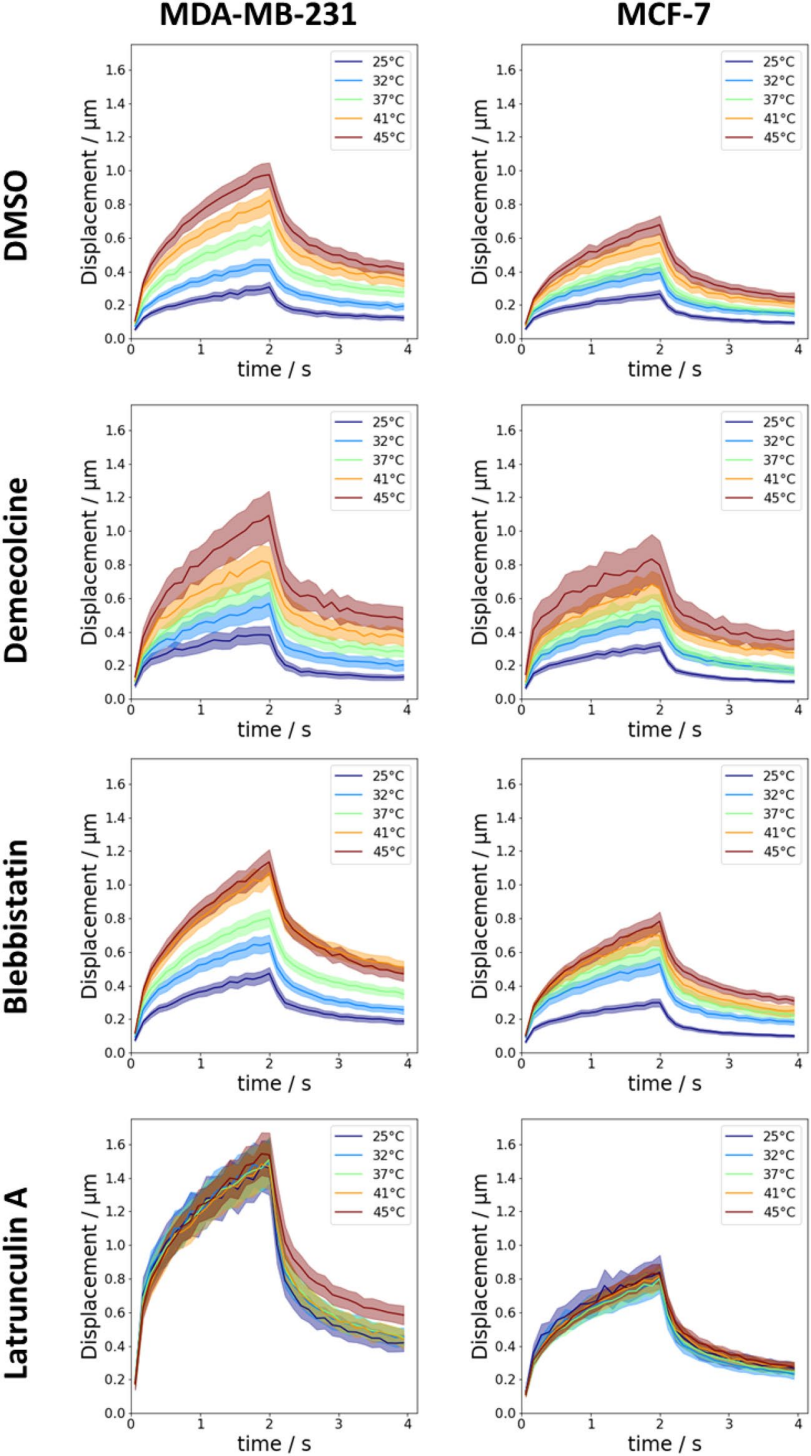
Averaged displacement curves of MDA-231 and MCF-7 cells after pharmacological intervention with different inhibitors targeting specific components of the cytoskeleton. Microtubules were depolymerized by treatment with 0.08 µM Demecolcine. Myosin-II was inhibited by treatment with 50 µM Blebbistatin. Actin polymerization was inhibited by treatment with 0.25 µM Latrunculin A. The control group was treated with an equal volume of the inhibitor solvent, in this case DMSO.

After treatment of the cells with the respective drugs, the temperature dependent measurements from above were repeated. The averaged displacement curves are presented in Fig. 3. Irrespective of treatment with drugs or increase in temperature, the displacement curves of all cells still followed a weak power law. The relative change of the creep compliance and cell fluidity from 25 to 45 °C of MDA-MB-231 and MCF-7 cells is summarized in Table 1 for the different tested inhibitors.

**Table 1:** Relative changes of creep compliance J_0_ and fluidity β from 25 to 45 °C for MDA-MB-231 and MCF-7 cells after treatment with different inhibitors.

Inhibitor	MDA-MB-231		MCF-7	
	J_0_^45°C^/J_0_^25°C^	β^45°C^/β^25°C^	J_0_^45°C^/J_0_^25°C^	β^45°C^/β^25°C^
Control	3.1 ± 0.7	1.2 ± 0.1	2.4 ± 0.6	1.4 ± 0.1
Blebbistatin	2.3 ± 0.5	1.2 ± 0.1	2.4 ± 0.6	1.4 ± 0.1
Demecolcine	2.5 ± 1.0	1.6 ± 0.2	2.6 ± 0.8	1.0 ± 0.2
Latrunculin A	1.0 ± 0.3	1.3 ± 0.1	1.0 ± 0.3	1.2 ± 0.1

### Comparison of temperature effect on cells for each drug individually

**Figure 4:**
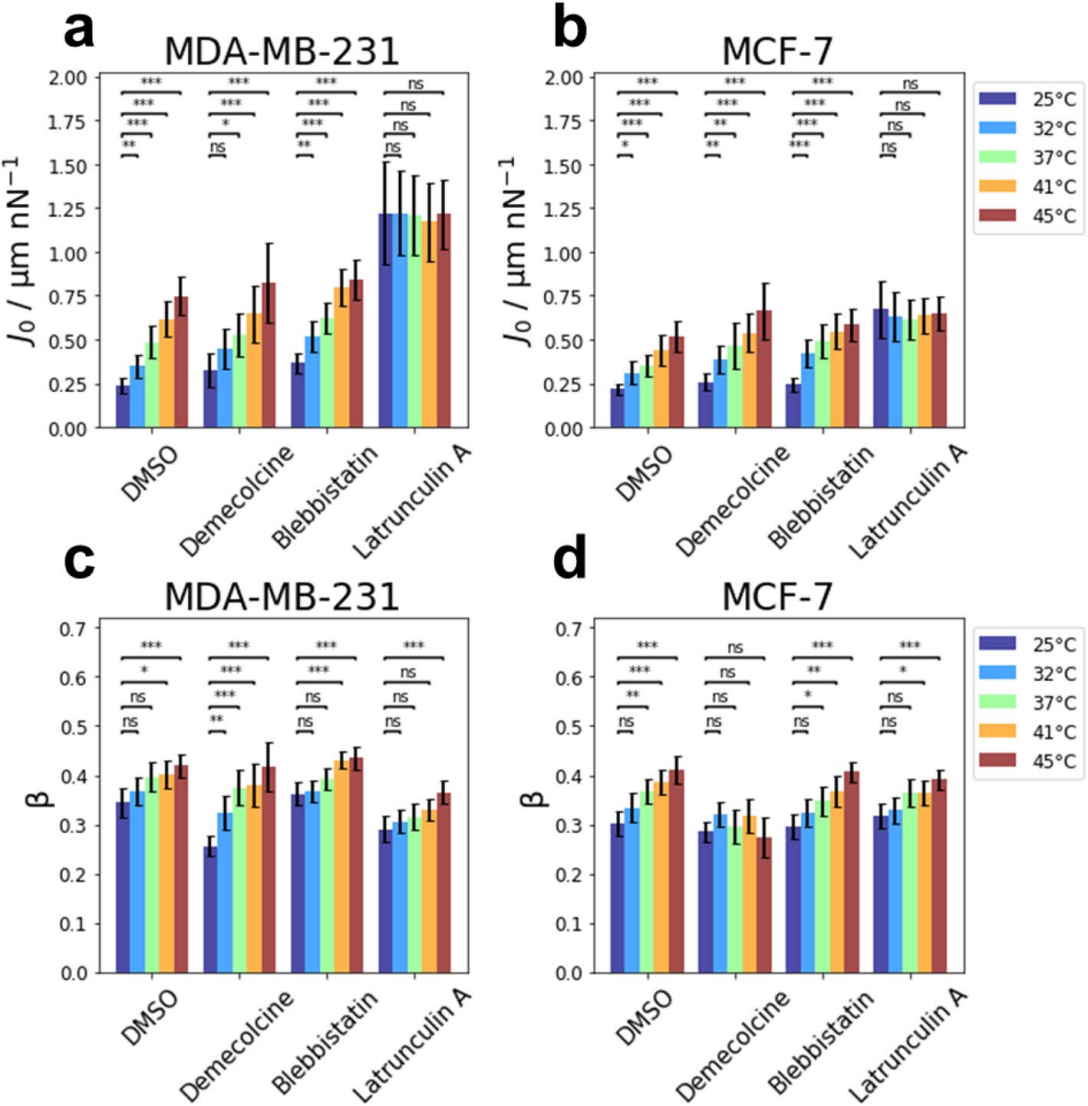
Comparison of the temperature dependence of mechanical properties for MDA-MB-231 and MCF-7 cells after pharmacological intervention. Influence of temperature on the creep compliance of (**a**) MDA-MB-231 cells and of (**b**) MCF-7 cells after treatment with Demecolcine, Blebbistatin and Latrunculin A. Effect of temperature on the cell fluidity β of (**c**) MDA-MB-231 and (**d**) MCF-7 cells after treatment with the afore mentioned inhibitors.

The effect of an increase in temperature on the creep compliance of MDA-MB-231 cells treated with Demecolcine, Blebbistatin and Latrunculin A, as well as the control group incubated with DMSO is presented in Fig. 4a. MDA-MB-231 cells supplemented with DMSO as a control group displayed a significant increase in compliance from 25 to 45 °C similar to untreated MDA-MB-231 cells (see Fig. 2). The control cells supplemented with DMSO experienced an increase in creep compliance by a factor of 3.1 ± 0.7 from 25 to 45 °C. The compliance of MDA-MB-231 cells treated with Demecolcine significantly increased by a factor of 2.5 ± 1.0 as the temperature was raised from 25 to 45 °C. Similarly, for Blebbistatin-treated MDA-MB-231 cells, a significant (2.3 ± 0.5)-fold increase in the compliance was observed from 25 to 45 °C. For the three tested substances described above (DMSO, Demecolcine, Blebbistatin) the increase in compliance with temperature appears to increase linearly with temperature in the investigated temperature range. However, the compliance of MDA-MB-231 cells treated with Latrunculin A was not affected by an increasing temperature and remained roughly constant. No significant changes were observed in their compliance between 25 and 45 °C (factor 1.0 ± 0.3). The relative changes of the creep compliance from 25 to 45 °C are summarized in Table 1.

The effect of an increase in temperature from 25 to 45 °C for MCF-7 cells treated with different drugs is presented in Fig. 4b. The control group of MCF-7 cells incubated with DMSO experienced a significant increase in compliance that roughly followed a linear relation. At the elevated temperature of 45 °C these MCF-7 cells displayed a (2.4 ± 0.6)-fold higher compliance compared to MCF-7 cells at 25 °C.

For MCF-7 cells that had their microtubule depolymerized by treatment with Demecolcine, a significant increase in compliance was observed as the temperature increased. The compliance increased linearly by a factor of 2.6 ± 0.8 from 25 to 45 °C. The compliance of MCF-7 cells treated with Blebbistatin increased by a factor of 2.4 ± 0.6 from 25 to 45 °C. In contrast to MCF-7 cells treated with Demecolcine, or the untreated cells, this increase in compliance did not follow a linear relation. Similar to MDA-MB-231 cells treated with Latrunculin A, MCF-7 cells treated with Latrunculin A did not experience a significant change in their creep compliance with increasing temperature (factor 1.0 ± 0.3). For all tested inhibitors, the relative changes of the creep compliance for MCF-7 cells are presented in Table 1.

The response of the cell fluidity to an increase in temperature on MDA-MB-231 cells treated with different inhibitors targeting specific cytoskeletal components is presented in Fig. 4c. The cell fluidity for the control group of MDA-MB-231 cells incubated with DMSO displayed the same response in temperature increase as the MDA-MB-231 cells measured in pure medium. Thus, a significant increase by a factor of 1.2 ± 0.1 was observed when the temperature was increased from 25 to 45 °C. MDA-MB-231 cells treated with Demecolcine at 45 °C displayed a significantly higher cell fluidity compared to MDA-MB-231 cells treated with Demecolcine at 25 °C (by a factor of 1.6 ± 0.2). For temperatures between 25 and 45 °C, a gradual increase in cell fluidity was observed for MDA-MB-231 cells after treatment with Demecolcine. Similarly, MDA-MB-231 cells treated with Blebbistatin at 45 °C displayed a significantly higher cell fluidity compared to those treated with Blebbistatin at 25 °C. However, the increase in fluidity was less drastic, as the cell fluidity only increased by a factor of 1.2 ± 0.1. The increase of cell fluidity only becomes significant above 37 °C when compared to that measured at 25 °C.

After treatment with Latrunculin A, MDA-MB-231 cells displayed an increase in cell fluidity by a factor of 1.3 ± 0.1 from 25 to 45 °C. This increase is a lot slower compared to the increase in cell fluidity of MDA-MB-231 cells treated with either Demecolcine or Blebbistatin. All changes in the fluidity from 25 to 45 °C of MDA-MB-231 cells are given in Table 1.

The effect of different drugs on the cell fluidity of MCF-7 is displayed in Fig. 4d for temperatures between 25 and 45 °C. The cell fluidity of MCF-7 supplemented with DMSO as a control displayed the same response to an increase in temperature as untreated cells (see Fig. 2). For these cells, a significant increase in cell fluidity by a factor of 1.4 ± 0.1 was observed from 25 to 45 °C. After treatment with Demecolcine, MCF-7 cells did not display a significant change in their cell fluidity. In the investigated temperature range between 25 and 45 °C, the cell fluidity of Demecolcine-treated MCF-7 cells remained roughly constant with a (1.0 ± 0.2)-fold change from 25 to 45 °C.

MCF-7 cells treated with Blebbistatin displayed a gradual and significant increase in cell fluidity from 25 to 45 °C by a factor of 1.4 ± 0.1. In contrast to the compliance, cell fluidity of MCF-7 cells treated with Latrunculin A did show a (1.2 ± 0.1)-fold increase from 25 to 45 °C. Similar to MDA-MB-231 cells, this increase is slower compared to the control or MCF-7 cells treated with either Demecolcine or Blebbistatin. The relative changes of the cell fluidity from 25 to 45 °C of MCF-7 cells are summarized in Table 1.

### Comparison of drug effect for each temperature

**Figure 5:**
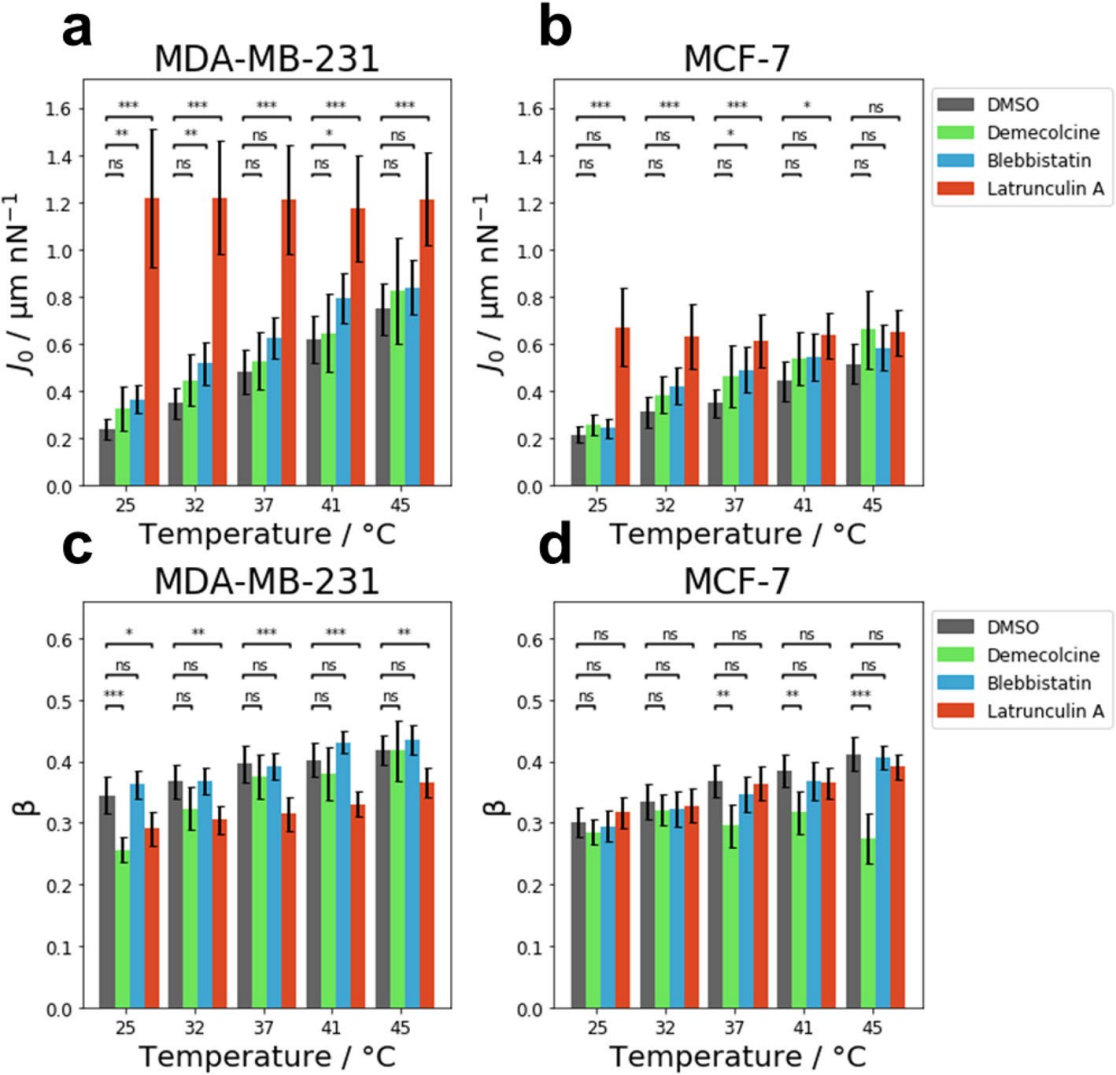
Comparison of the effect of treatment with Demecolcine, Blebbistatin and Latrunculin A on the mechanical properties for MDA-MB-231 and MCF-7 cells at different temperatures. Creep compliance of (**a**) MDA-MB-231 and (**b**) MCF-7 cells was not affected by Demecolcine at any temperature, while Latrunculin A and Blebbistatin affected the creep compliance of both cell lines. Cell fluidity β of (**c**) MDA-MB-231 cells and (**d**) MCF-7 cells was not affected by Blebbistatin at any temperature. Latrunculin A only affected the cell fluidity of MDA-MB-231 cells but not MCF-7 cells. Demecolcine displayed a different temperature dependent effect on the cell fluidity of MDA-MB-231 and MCF-7 cells.

For MDA-MB-231 cells, the effect of tested substances (DMSO (control), Demecolcine, Blebbistatin, Latrunculin A) on the creep compliance is presented in Fig. 5a. For all temperatures in the range from 25 to 45 °C, the creep compliance of MDA-MB-231 cells treated with Demecolcine did not differ significantly compared to untreated cells. MDA-MB-231 cells treated with Blebbistatin displayed a significantly higher creep compliance at temperatures 25 °C, 32 °C and 41 °C. However, this difference was most pronounced at lower temperatures (25 °C and 32 °C). At the highest investigated temperature (45 °C), no significant softening due to treatment with Blebbistatin was observed for MDA-MB-231 cells. Depolymerization of actin by treatment with Latrunculin A had the strongest effect on creep compliance of all the tested drugs in the investigated temperature range. For MDA-MB-231 cells, Latrunculin A had a significant softening effect at all temperatures compared to untreated cells.

The effect of the different drugs on the compliance of MCF-7 cells at each temperature is presented in Fig. 5b. Similar to MDA-MB-231 cells, Demecolcine did not affect the compliance of MCF-7 significantly. Blebbistatin-treated MCF-7 cells displayed a slightly elevated creep compliance in the investigated temperature range. However, this increase in compliance was only significant at a temperature of 37 °C. MCF-7 cells treated with Latrunculin A displayed an elevated creep compliance at all temperatures compared to untreated cells. This difference was most pronounced at 25 °C. At 45 °C the creep compliance of untreated cells was almost at the same level as the creep compliance of Latrunculin A and did not show a significant difference.

In Fig. 5c, the cell fluidity of MDA-MB-231 cells treated with Demecolcine, Blebbistatin and Latrunculin A, as well as the control with DMSO were compared at each of the investigated temperatures. MDA-MB-231 cells treated with Demecolcine displayed a significantly reduced cell fluidity at 25 °C compared to untreated cells. However, at higher temperatures (32 °C, 37 °C, 41 °C and 45 °C) treatment with Demecolcine did not affect the cell fluidity significantly. For MDA-MB-231 cells treated with Blebbistatin, no significant change in cell fluidity was observed at any of the investigated temperatures when compared to untreated cells. Latrunculin A induced a significant decrease in cell fluidity at all temperatures in the investigated temperature range.

For MCF-7 cells, the effect of the investigated substances on the cell fluidity at each of the temperatures in the range between 25 and 45 °C is presented in Fig. 5d. The cell fluidity of MCF-7 cells treated with Demecolcine at 25 °C and 32 °C did not differ significantly from the cell fluidity of untreated MCF-7 cells at the respective temperature. However, MCF-7 cells treated with Demecolcine at higher temperatures (37 °C, 41 °C and 45 °C) displayed a significantly lower cell fluidity when compared to untreated cells. The difference in the cell fluidity between untreated and Demecolcine-treated cells was most pronounced at the highest temperature (45 °C). Neither treatment with Blebbistatin nor Latrunculin A affected the cell fluidity of MCF-7 cells at the given temperature when compared to untreated cells at the same temperature.

## Discussion

Temperature is an external factor that may greatly influence the behavior of cells. Many vital processes of the cell, such as chemical reactions or the functionality of several proteins, are dependent on temperature^22^. Likewise, temperature also influences the organization of the cytoskeleton and therefore impacts its mechanical properties even for small temperature changes^12,19,22^. So far, the mechanism behind the changes in cellular compliance in response to increasing and decreasing temperatures is not well understood^13,15,19^.

Commonly used heating systems in experiments with living cells include heating chambers^15,53^, a heated microscopic stage^54^, or a heated water bath^22^. In this work, temperature was precisely controlled by a heatable incubation chamber that encloses the entire measurement setup including the magnetic tweezer needle. Thus, the incubation chamber provides stable environmental conditions during the entire measurement in the temperature-range of 25 °C to 45 °C.

In order to understand the process of hyperthermia, we employed two representative human breast cancer cell lines and analyzed their cell mechanics in response to temperature changes and pharmacological interventions. MCF-7 as well as MDA-MB-231 cells displayed a pronounced softening and fluidization with increasing temperature. For both cell lines, the compliance increased linearly with temperature. This is in line with an earlier study on HL60/S4 myeloid precursor cells measured by an optical stretcher^15^. Therein, the linear increase has been linked to the network elasticity of densely crosslinked gels of semiflexible polymers^15,55^. Several other studies detected similar results, i.e. a gradual decrease of the elastic modulus with temperature^1,13,15–22^. In contrast, some studies^12–14^ reported an increase in the elastic modulus with increasing temperatures. Hence, the dependence is still not clearly understood. These differences may be explained by a cell-type specific response of cells to alterations in temperature^13,19^. However, as different cell types differ in their structure and functionality it is not clear if the observed effect of temperature induced softening and fluidization are universal properties of cells. Different cell types may display opposite effects, e.g. due to a more active force generation that increases the cytoskeletal prestress and thus lowers compliance at higher temperatures^17,19^.

After investigating the response of the creep compliance and fluidity of MDA-MB-231 and MCF-7 cells to an increase in temperature, a significant difference in the behavior of these two cell lines was observed. Both cell lines experienced a temperature induced softening and fluidization. MDA-MB-231 cells were consistently softer and more fluid-like compared to MCF-7 cells, irrespective of temperature. This is consistent with other studies that investigated the mechanical properties of these cells at 37 °C^30,31,35,37,39,56^. However, the average creep compliance of MDA-MB-231 cells rose much faster than that of MCF-7 cells. One explanation may be that the two investigated cell lines possess a different cytoskeletal architecture. Hence, it is conceivable that the different responses are due to these differences in structure^13^. It has been proposed that differences in the mechanical properties of MCF-7 and MDA-MB-231 cells are linked to differences in their cytoskeletal organization^30,31,39^. In an earlier study, we found that the cytoskeletal structure of MDA-MB-231 cells differs from that of MCF-7 cells. Compared to MCF-7 cells, the thickness of actin stress fibers was reduced in MDA-MB-231^31,36^. Similarly, in^35^ it was reported that the cell body of MCF-7 cells exhibits a homogeneous distribution of stiffness and fluidity, while MDA-MB-231 cells display local variances in their stiffness and fluidity. These differences in the mechanical properties are also reflected in morphological differences^30^. Thus, the different cell mechanical phenotype of the two cell types can be explained through their diverse cytoskeletal structure.

Changes in cellular mechanics due to elevated temperatures have been linked to the reorganization of cytoskeletal structures^1,13,16^. Since the cytoskeleton plays a crucial role in mechanical interactions of cells with their environment^31,39^, we postulate that the observed difference in the change of mechanical parameters between MCF-7 and MDA-MB-231 cells also stems from a difference in the cytoskeletal structure between the two cell lines. Different molecular mechanics may contribute to the reported changes in cellular mechanics with temperature^18^.

The power law used to analyze the creep compliance of the cells does not allow specific cytoskeletal components to be linked to the derived properties of compliance and fluidity. However, it has been reported that power law rheology is a fundamental property of cells^47^. Thus, it is possible to describe the overall mechanical behavior of cells employing this power law^33,47,48^, which in turn permits the examination of the influence that individual components of the cytoskeleton exhibit on this overall mechanical behavior.

In order to investigate the contribution of actin, myosin and microtubules on the temperature induced changes in the mechanical properties, these cytoskeletal components were specifically targeted with inhibitors. The polymerization of microtubule was inhibited by treatment with Demecolcine. For MDA-MB-231 and MCF-7 cells, treatment with Demecolcine did not affect the compliance in a significant way at any temperature. However, at lower temperatures (below 37 °C) the cell fluidity of MDA-MB-231 cells was significantly reduced compared to untreated cells. For MCF-7 cells, on the other hand, Demecolcine treatment actually abolished the temperature dependency of the cell fluidity, as the cell fluidity remained roughly constant in the investigated temperature range. Both cell lines still displayed a significant temperature induced softening after treatment with Demecolcine.

Earlier studies investigating the effect of temperature on microtubules found temperature sensitive effects on the assembly and disassembly of microtubules^20,57,58^ in the range of temperatures investigated in this work. Depending on the temperature, it can either increase or decrease the stability of microtubules^58^. Additionally, the flexural rigidity of microtubules may be sensitive to changes in temperature^58,59^. However, the contribution of the microtubules to the temperature induced changes in cell stiffness have been reported to be insignificant in cells^19^. There, stabilizing the microtubules with taxol had no significant effect on the temperature dependence compared to untreated cells^19^. Finally, inhibition of microtubules did not abolish the temperature dependent softening of cells. However, the effect was attenuated as evident from the reduced relative change in creep compliance from 25 to 45 °C. Our results of measurements on living cells with impaired microtubule polymerization support the results presented in^19^. Therefore, we conclude that microtubules play only a minor role, if any at all, in the elastic response to an increased temperature of the investigated cell lines. However, Demecolcine treatment affected the response of the cell fluidity to an increase in temperature in both cell lines, thus indicating that microtubules may contribute to the viscous response of cells to changes in temperature.

Myosin-II is a molecular motor that has been linked directly to the temperature-induced changes in the mechanical properties of cells^14,19^. The forces generated by myosin motors play an important role in the mechanics of the cytoskeleton, as they result in the sliding of filaments past each other^19^. The activity of myosin has been seen to increase with temperature^60,61^. For high myosin activities, the cytoskeleton can therefore be softened and fluidized locally^19^. When myosin is inhibited by pharmacological intervention, the susceptibility of cells to changes in temperature is significantly reduced^19^. However, in another study^14^ it has been reported that the increased myosin-activity actually increases the contractility and thus lowers the compliance in fibroblasts.

Our results on the measurement of cellular compliance and fluidity of MCF-7 and MDA-MB-231 cells did show that the effect of myosin-II inhibition on cellular mechanics depends on the ambient temperature. Although cells treated with Blebbistatin experienced a temperature induced softening and fluidization, the effect of Blebbistatin treatment compared to untreated cells was most prominent at lower temperatures for MDA-MB-231 cells and at intermediate temperatures for MCF-7 cells. These results combined with the results presented in^14,19^ indicate a cell-type dependent response of myosin in cells to temperature changes. Although cells treated with Blebbistatin experienced an increase in creep compliance with temperature, this response was attenuated compared to untreated cells. The relative change of cells treated with Blebbistatin was similar to that of cells treated with Demecolcine. This suggests that myosin filaments and microtubules contribute similarly to the temperature-dependent softening of MDA-MB-231 and MCF-7 cells.

Filamentous actin is one of main structural components of the cytoskeleton and plays an essential role in the mechanical response of cells to forces and deformation^30^. Disrupting the actin cytoskeleton has been shown to significantly increase the compliance of cells both in this work and earlier studies^20,31^. Due to its importance in the mechanical integrity of cells, the temperature dependent behavior of cells is most likely tightly coupled to the temperature induced structural changes of the actin cytoskeleton^13,17,19,62^.

After treatment with Latrunculin A, an agent that inhibits the polymerization of actin, both MCF-7 and MDA-MB-231 cells displayed a significantly increased creep compliance. However, cells treated with Latrunculin A did not display significant differences in their creep compliance when heated up to 45 °C. Since the creep compliance of cells treated with Latrunculin A, i.e. cells with a disrupted actin cytoskeleton, was not affected by an increase in temperature, the observed temperature induced softening in the initial experiments can be mainly attributed to the response of the actin cytoskeleton. In^17^, the actin polymerization of cells has been inhibited using cytochalasin D. Cells treated with this drug remained unaffected by changes in temperature. These findings support the idea presented in this work that the temperature dependent changes in compliance and fluidity are mainly carried by the actin cytoskeleton.

The effect of Latrunculin A on the fluidity of cells differed between the two cell lines. While MCF-7 cells were not affected by treatment with Latrunculin A, MDA-MB-231 cells displayed a lower fluidity after the treatment. Both cell lines experienced a temperature induced increase in fluidity after the treatment with Latrunculin A.

A possible explanation for the observed increase in cell fluidity may be a temperature induced unbinding of weak mechanical bonds that crosslink the actin cytoskeleton of the cells. Many earlier numerical^63,64^ and experimental^35,62,65–68^ studies reported that the viscosity (i.e. fluidity in this work) of crosslinked networks, such as the actin network of the cytoskeleton, is dominated by the unbinding of weak non-covalent bonds. Similar results have been found for reconstituted microtubule networks crosslinked by biotin-streptavidin bonds^69^. The unbinding of these bonds is responsible for typical viscoelastic properties of these networks, such as the continuous creep when an external force is applied^64,69,70^. Densely crosslinked networks generally show a higher elastic modulus and a more solid-like behavior^63,69,70^.

At elevated temperatures the dissociation rate of transient crosslinks in a network increases^62,66–68,71–73^. Due to the increased unbinding at higher temperatures, a transition from a crosslinked to an entangled network can be observed^65^. Experimental studies have shown that the increased unbinding rate of crosslinks in a network leads to a softening and fluidization^62,66–68,70,72^.

The temperature induced cell softening and fluidization observed in this work may therefore be attributed to an increased unbinding of cytoskeletal crosslinks at higher temperatures^14,15,19,35^. Due to the increased unbinding rate of the cytoskeletal crosslinks, the cytoskeleton is destabilized. Therefore, the network becomes more compliant to external stresses. The more rapid unbinding of crosslinks at higher temperatures leads to a more viscous-like behavior of the network as filaments can slide past each other more easily to dissipate stress^15,35,62^*.*

The temperature induced disorganization of actin networks in vivo may be attenuated by the presence of small heat shock proteins^74^. However, the expression of these proteins may be dysregulated in cancerous cells^23,75^, thus providing a possible explanation for the observed differences in the thermorheological response of MCF-7 and MDA-MB-231 cells.

Our findings outline the importance of a proper temperature control in measurements of living cells, as even small changes in temperature can significantly affect the measured mechanical properties. This is particularly important in experiments that involve the treatment of cells with various inhibitors. Our results show that certain inhibitors, such as Demecolcine or Blebbistatin, may influence the mechanical properties of cells to different degrees depending on the ambient temperature. In conclusion, the main contributor to the temperature dependence of cellular mechanics seems to be the actin cytoskeleton, while microtubules and myosin seem to play only a minor role.

## Supplementary Information

Supplementary Figure S1.

Supplementary Video 1.

Supplementary Video 2.

Supplementary Video 3.

Supplementary Video 4.

Supplementary Video 5.

Supplementary Video 6.

Supplementary Video 7.

Supplementary Video 8.

## Data Availability

The datasets generated during and/or analyzed during the current study are available from the corresponding author on reasonable request.
