# Supplementary material for: Cell mechanical properties of human breast carcinoma cells depend on temperature
Source: Sci Rep. 2021 May 24;11:10771. doi: 10.1038/s41598-021-90173-y (PMC8144563; doi:10.1038/s41598-021-90173-y)
Supplement: Supplementary file 1 — Supplementary Figure S1. [file 41598_2021_90173_MOESM1_ESM.pdf]

Supplementary Information

Cell mechanical properties of human breast carcinoma cells depend on temperature

Christian Aermes, Alexander Hayn, Tony Fischer and Claudia Tanja Mierke*

Faculty of Physics and Earth Science, Peter Debye Institute of Soft Matter Physics, Biological Physics Division, University of Leipzig, Linnéstr. 5, 04103 Leipzig, Germany

*Corresponding author:
Claudia Tanja Mierke, Faculty of Physics and Earth Science, Peter Debye Institute of Soft Matter Physics, Biological Physics Division, University of Leipzig, Linnéstr. 5, 04103 Leipzig, Germany, Tel.: ++ 49-341-9732541, Fax: ++ 49-341-9732599
E-Mail: claudia.mierke@uni-leipzig.de

Supplementary Figure 1

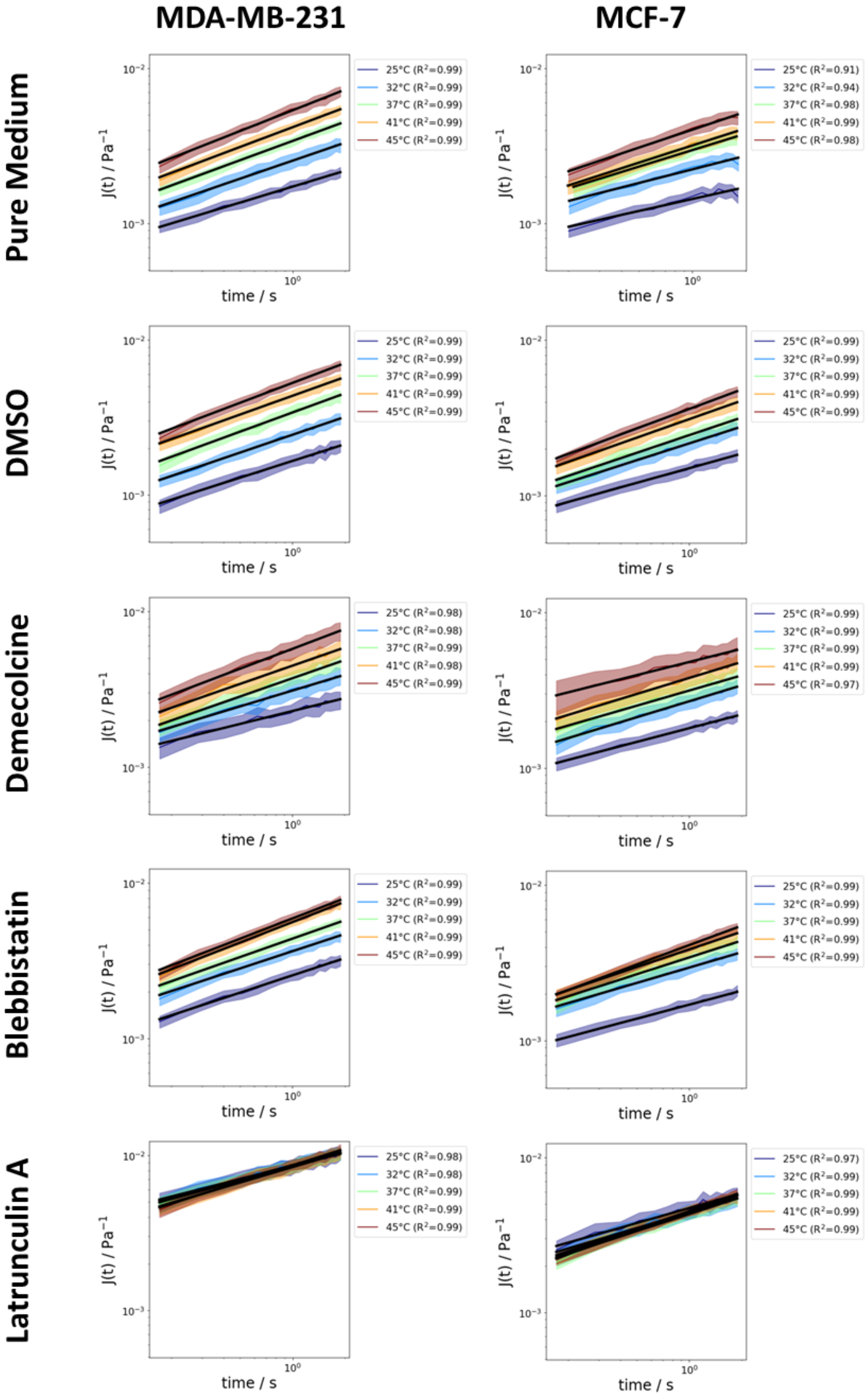

Supplementary Figure 1: Creep compliance curves of MDA-MB-231 and MCF-7 cells in response to the application of a 1 nN force pulse. Curves are displayed in a double logarithmic scale and fitted with a weak power law. The goodness of the fit is given by R^2. The power law fits the experimental curves well for both breast cancer cell lines and all investigated pharmacological treatments, as evident from the high R^2-values.

Supplementary Video 1: Video of a representative MDA-MB-231 cell measured at the beginning of an experiment recorded at 25°C. The coupled bead moves in response to the application of a constant force of 1 nN. Scale bar is 10 μm.

Supplementary Video 2: Video of a representative MCF-7 cell measured at the beginning of an experiment recorded at 25°C. The coupled bead moves in response to the application of a constant force of 1 nN. Scale bar is 10 μm.

Supplementary Video 3: Video of a representative MDA-MB-231 cell measured at the end of an experiment taken at 25°C. The coupled bead moves in response to the application of a constant force of 1 nN. Scale bar is 10 μm.

Supplementary Video 4: Video of a representative MCF-7 cell measured at the end of an experiment taken at 25°C. The coupled bead moves in response to the application of a constant force of 1 nN. Scale bar is 10 μm.

Supplementary Video 5: Video of a representative MDA-MB-231 cell measured at the beginning of an experiment taken at 45°C. The coupled bead moves in response to the application of a constant force of 1 nN. Scale bar is 10 μm.

Supplementary Video 6: Video of a representative MCF-7 cell measured at the beginning of an experiment taken at 45°C. The coupled bead moves in response to the application of a constant force of 1 nN. Scale bar is 10 μm.

Supplementary Video 7: Video of a representative MDA-MB-231 cell measured at the end of an experiment taken at 45°C. The coupled bead moves in response to the application of a constant force of 1 nN. Scale bar is 10 μm.

Supplementary Video 8: Video of a representative MCF-7 cell measured at the end of an experiment taken at 45°C. The coupled bead moves in response to the application of a constant force of 1 nN. Scale bar is 10 μm.
